# Optimal Cutoff Values of WHO-HPQ Presenteeism Scores by ROC Analysis for Preventing Mental Sickness Absence in Japanese Prospective Cohort

**DOI:** 10.1371/journal.pone.0111191

**Published:** 2014-10-23

**Authors:** Tomoko Suzuki, Koichi Miyaki, Yasuharu Sasaki, Yixuan Song, Akizumi Tsutsumi, Norito Kawakami, Akihito Shimazu, Masaya Takahashi, Akiomi Inoue, Sumiko Kurioka, Takuro Shimbo

**Affiliations:** 1 Department of Clinical Research and Informatics, National Center for Global Health and Medicine, Tokyo, Japan; 2 Department of Public Health, Kitasato University School of Medicine, Kanagawa, Japan; 3 Department of Mental Health, Tokyo University Graduate School of Medicine, Tokyo, Japan; 4 Health Administration and Psychosocial Factor Research Group, National Institute of Occupational Safety and Health, Kanagawa, Japan; 5 Department of Mental Health, Institute of Industrial Ecological Sciences, University of Occupational and Environmental Health, Fukuoka, Japan; 6 Department of Health Policy and Management, University of Occupational and Environmental Health, Fukuoka, Japan; United (Osaka U, Kanazawa U, Hamamatsu U Sch Med, Chiba U and Fukui U) Graduate School of Child Development, Japan

## Abstract

**Objectives:**

Sickness absence due to mental disease in the workplace has become a global public health problem. Previous studies report that sickness presenteeism is associated with sickness absence. We aimed to determine optimal cutoff scores for presenteeism in the screening of the future absences due to mental disease.

**Methods:**

A prospective study of 2195 Japanese employees from all areas of Japan was conducted. Presenteeism and depression were measured by the validated Japanese version of the World Health Organization Health and Work Performance Questionnaire (WHO-HPQ) and K6 scale, respectively. Absence due to mental disease across a 2-year follow-up was surveyed using medical certificates obtained for work absence. Socioeconomic status was measured via a self-administered questionnaire. Receiver operating curve (ROC) analysis was used to determine optimal cutoff scores for absolute and relative presenteeism in relation to the area under the curve (AUC), sensitivity, and specificity.

**Results:**

The AUC values for absolute and relative presenteeism were 0.708 (95% CI, 0.618–0.797) and 0.646 (95% CI, 0.546–0.746), respectively. Optimal cutoff scores of absolute and relative presenteeism were 40 and 0.8, respectively. With multivariate adjustment, cohort participants with our proposal cutoff scores for absolute and relative presenteeism were significantly more likely to be absent due to mental disease (OR = 4.85, 95% CI: 2.20–10.73 and OR = 5.37, 95% CI: 2.42–11.93, respectively). The inclusion or exclusion of depressive symptoms (K6≥13) at baseline in the multivariate adjustment did not influence the results.

**Conclusions:**

Our proposed optimal cutoff scores of absolute and relative presenteeism are 40 and 0.8, respectively. Participants who scored worse than the cutoff scores for presenteeism were significantly more likely to be absent in future because of mental disease. Our findings suggest that the utility of presenteeism in the screening of sickness absence due to mental disease would help prevent such an absence.

## Introduction

Long-term sickness absence from work due to mental disease has become a global public health problem [1]. For example, Sickness absence due to mental disease, particularly stress-related mental disease, has increased in a number of European countries over the last 20 years [2]. In Japan, a survey of government officials found that mental and behavioral disorders were the most frequent causes of long-term absence, constituting 64.6% of all long-term absences due to illness. Similarly, over the past decade, mental and behavioral disorders have been ranked first among causes of long-term illness-related absences [3].

Employee illness can result in lost productivity in the form of absenteeism and presenteeism. “Absenteeism” refers to an employee’s time away from work due to illness or disability. “Presenteeism,” which constitutes a kind of job performance, refers to the decrease in productivity in employees who are present but not functioning at full capacity because of illness or other medical conditions [4], [5]. Furthermore, absolute presenteeism can be calculated as the difference between the score for self over the past 28 days and the score for the average worker in the same job. A relative presenteeism score can be computed as the ratio of self versus other scores [6].

Risk factors for presenteeism or absence (absenteeism) [7], [8], and a relationship between sickness presenteeism and sickness absence have been observed. Taloyan et al. reported that sickness presenteeism predicts suboptimal self-rated health and sickness absence two years later [9]. Hansen et al. reported that presenteeism is associated with absence due to long-term sickness, and that participants who had gone to work ill more than six times in the year prior to baseline had a 74% higher risk of an absence lasting more than 2 months [10]. Bergström et al. reported that sickness presenteeism on more than five occasions during the baseline year was a statistically significant risk factor for a future illness-related absence of more than 30 days [11]. We previously found a relationship between presenteeism and greater risk of absences due to mental disease in a large-scale cohort of Japanese workers (submitted for publication). Moreover, we indicated that measurement of presenteeism could be used to predict absence risk: specifically, optimal cutoff scores for presenteeism could be established as a means of identifying high-risk employees. In public health, various prediction models have been developed to predict the future occurrence of disease and to target preventive interventions at high-risk subjects. However, to date, there is no optimal cutoff score for presenteeism that could predict the likelihood of absence due to mental disease. Recently, the Japanese study of Health, Occupation and Psychosocial factor related Equity (J-HOPE study [12], [13]) was performed to develop and expand research aimed at elucidating the mechanisms underlying social disparities in health and to establish control measures. The goal of the current study, a part of J-HOPE, was to determine an optimal cut-off value for presenteeism in the screening of the absence due to mental disease in the future.

## Materials and Methods

### Participants

**Figure 1:**
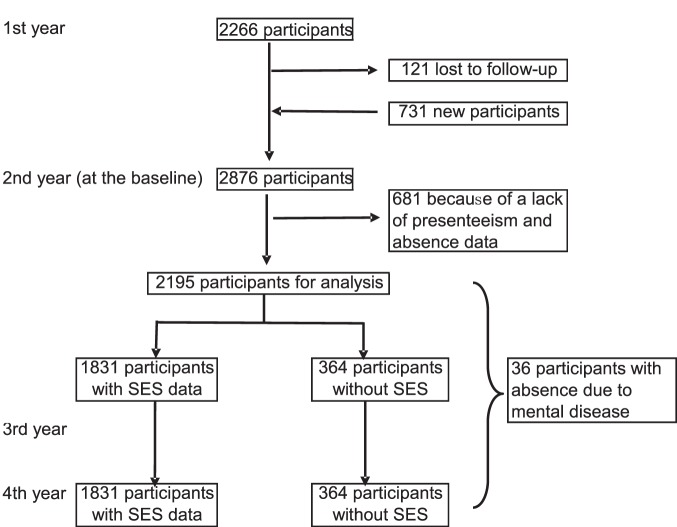
Flowchart for our cohort study. SES: Socioeconomic status.

The present longitudinal study was based on data obtained from a survey conducted for our occupational cohort study on social class and health, which was supported by a grant from the Ministry of Education, Culture, Sports, Science, and Technology, Japan. Employees of a major Japanese manufacturing company (headquartered in Kyoto, with 11 other major offices throughout Japan) were recruited. All workers were invited to participate, and 2266 agreed (response rate: 90.1%; age range: 21–65 years; 241 women and 2025 men) in the first year (2010), 2876 participants agreed in the second year (2011), and 2624 participants agreed in the third year (2012). Of the 2876 participants in the second year, 731 participants were new and 2145 participants were the same one who agreed in the first year. Of the initial 2876 participants in the second year, 681 participants for whom presenteeism or absenteeism data were not available at the time were excluded from the analyses. Thus, analyses reported in this study were restricted to the 2195 participants with data on presenteeism in the second year (at baseline) and sickness absences due to mental disease or due to non-mental disease across a 2-year follow-up. Eligible participants (n = 2195) and non-eligible participants (n = 681) did not differ in gender, age, income, managerial job, length of service, education level, smoking habits and exercise in spare time at baseline. But there were statistical significant differences between the groups with K6 score (5.4±0.1 vs. 4.7±0.2 [mean ± SE]), number of people in the household (2.9±0.0 vs. 2.7±0.1), and drinking habits (drink approximately every day, 31.9% vs. 38.6%). However, these differences had no medical significance. Absence dates for 10 of the 36 participants who had an absence due to mental disease were not identified. Therefore, the date was defined as the median of the follow-up period in order to define whether the absence dates were during 2 year follow up. Of the 2,195 participants included the analysis, socioeconomic data were not available for 364 participants (Fig. 1).

The J-HOPE study received ethical approval from the University of Tokyo Ethics Committee and the National Center for Global Health and Medicine Ethics Committee. All participants provided written informed consent before study enrollment. Job position, years of education, annual household income, number of people in the household, drinking habits, smoking habits, exercise during spare time, and length of service were assessed through a self-administered questionnaire. All the sickness absences were registered by the medical center of the participating company. Absences due to mental disease were confirmed through medical certificates for work absence. If the employee is absent more than 3 days, the illness must be confirmed by a medical certificate. Participants who had been absent for reasons other than mental disease comprised one group, while participants who had been absent due to mental disease comprised another group.

### Health and Work Performance Questionnaire

Job performance was assessed using the World Health Organization Heath and Work Performance Questionnaire (WHO-HPQ) [4]–[6] at baseline. The WHO-HPQ is a self-report instrument designed to estimate the workplace costs of health problems in terms of self-reported sickness absence and reduced job performance (presenteeism). We used the validated Japanese version of the WHO-HPQ short form, which we translated into Japanese and had independently back-translated by an native English speaker. The official version is available [14]. The HPQ measures presenteeism through the following two questions: “On a scale from 0 to 10, where 0 is the worst job performance anyone could have at your job and 10 is the performance of a top worker, how would you rate the usual performance of most workers in a job similar to yours?” and “Using the same 0–10 scale, how would you rate your overall job performance on the days you worked during the past four weeks?” The absolute presenteeism score was obtained by multiplying the participant’s response to the second question by 10. The relative presenteeism score was obtained by dividing the second response by the first response. Absolute presenteeism score ranged from 0 (total lack of performance during time on the job) to 100 (no lack of performance during time on the job). Relative presenteeism is a ratio of actual performance to possible performance (the performance of most workers in the same job) and ranged from 0.25 to 2.0, where 0.25 indicated the worst (25% or less of other workers’ performance) and 2.0 signified the best relative performance (200% or more of other workers’ performance). In other words, a low presenteeism score indicated poorer performance.

### Psychological distress

Depression was measured with the Japanese version of the K6 scale [15], [16] at baseline and after one year. The K6 scale consists of six items that address the frequency of psychological distress symptoms (e.g., “feeling so sad that nothing can cheer you up”) during the previous 30 days. The response options range from 0 (none of the time) to 4 (all of the time), with the possible total score ranging from 0 to 24. The scale’s internal reliability and validity, documented in previous research, were acceptable. In the present sample, Cronbach’s alpha coefficient (0.85 [17]) also met the acceptability criteria for both men and women. Depressive symptoms were defined as present when subjects had a K6 score ≥13, indicating severe mental illness [18].

### Statistical analysis

Differences in means between groups of the participants with and without absence due to mental disease were tested using *t* tests. Associations between categorical variables were tested using χ^2^ tests.

The sensitivity, specificity, and the area under the curve (AUC) of different cut-off values of absolute and relative presenteeism were calculated. The ROC curve graphically displays the trade-off between sensitivity and specificity and is useful in assigning the best cut-offs for clinical use [19], [20].

Multiple logistic regression analysis was performed to estimate the odds ratios (ORs) and 95% confidence intervals (CI) of absences due to mental disease or non-mental disease across a 2-year follow-up, using the optimal cutoff points of absolute or relative presenteeism scores at baseline as independent variables. Furthermore, multiple logistic regression analysis was performed to estimate the ORs and 95% CIs of depressive symptoms (K6 score ≥13) after one year for the optimal cutoff points of absolute or relative presenteeism scores at baseline. The first model was adjusted for age and gender; a second model was further adjusted for depressive symptoms (K6≥13) at baseline; and a third model was further adjusted for drinking habits (drink approximately every day or not), smoking habits (current smoker or not), education level (years), job position (managerial job or not), equivalent income (annual household income divided by the square root of the household number), and exercise in spare time (yes or no) at baseline. Two-tailed P-values of less than 0.05 were considered statistically significant. All analyses were conducted using SPSS (version 20 for Windows, IBM Inc., New York, USA).

## Results

The baseline characteristics of study participants grouped according to whether they had been absent due to mental disease across a 2-year period are presented in Table 1. Thirty-six participants (1.6%) were absent due to mental disease. Participants with a history of absence due to mental disease had significantly worse absolute and relative presenteeism scores. Absolute presenteeism score had a large effect size, while relative presenteeism score had a small effect size. Participants who were absent because of mental disease reported more depressive symptoms (K6≥13), although no differences were found with respect to the mean of K6 score**.** There were no associations between socioeconomic variables and absence.

**Table 1:** Participant characteristics by whether participants had been absent due to mental disease across a 2-year follow-up.

	n	All participants	Absence due to mental disease		
			−	+	P
No. of subjects for analysis		2195	2159 (98.4)	36 (1.6)	
Gender, Male, n (%)	2195	1955 (89.1)	1921 (89.0)	34 (94.4)	0.297
Job performance					
Absolute presenteeism score, mean ± SD	2195	57.3±18.4	57.5±18.3	42.5±20.6	<0.001*
Relative presenteeism score, mean ± SD	2157	1.0±0.3	1.0±0.3	0.9±0.4	0.006*
No. of subjects excluded for missing data		1831	1805 (98.6)	26 (1.4)	
Gender, Male, n (%)	1831	1633 (89.2)	1607 (89.0)	26 (100.0)	0.074
Age, mean ± SD	1831	43.2±9.4	43.2±9.5	44.7±8.2	0.407
Depressive symptoms					
K6 score, mean ± SD	1830	5.4±4.8	5.4±4.7	6.4±7.0	0.279
K6 score (≥13), n (%)	1830	149 (8.1)	144 (8.0)	5 (19.2)	0.037*
Socioeconomic status					
Income (10,000 yen/year)	1828	454.8±186.6	454.3±186.6	490.2±186.5	0.330
Job position, n (%)					
Managerial job	1820	439 (24.1)	430 (24.0)	9 (34.6)	0.208
Length of service (years), mean ± SD	1831	20.3±11.1	20.3±11.1	22.2±10.5	0.382
Number of people in the household (n), mean ± SD	1831	2.9±1.4	2.9±1.4	3.1±1.5	0.439
Education level (years), mean ± SD	1830	14.5±2.5	14.5±2.5	14.8±2.3	0.562
Drinking habits (drink approximately every day), n (%)	1831	706 (38.6)	697 (38.6)	9 (34.6)	0.677
Smoking habits (current smoking), n (%)	1828	545 (29.8)	535 (29.7)	10 (38.5)	0.332
Exercise in spare time (no exercise), n (%)	1827	1006 (55.1)	990 (55.0)	16 (61.5)	0.504

– : never absent due to mental disease; +: absent at least once due to mental disease; ΔK6 score (K6 score of the third year - K6 score of second year); income: equivalent income; *P<0.05; difference in proportions and means was assessed by using χ^2^ test and independent *t* test, respectively.

Participants who had experienced an absence due to non-mental disease had significantly worse absolute presenteeism scores than did participants who had not been absent because of non-mental disease, but relative presenteeism score did not differ between the two groups. Participants who had been absent because of non-mental disease had more depressive symptoms (K6≥13), were younger, and had a shorter length of service (data not shown).

### Optimal cutoff values of presenteeism scores for the prevention of mental sickness absence

**Figure 2:**
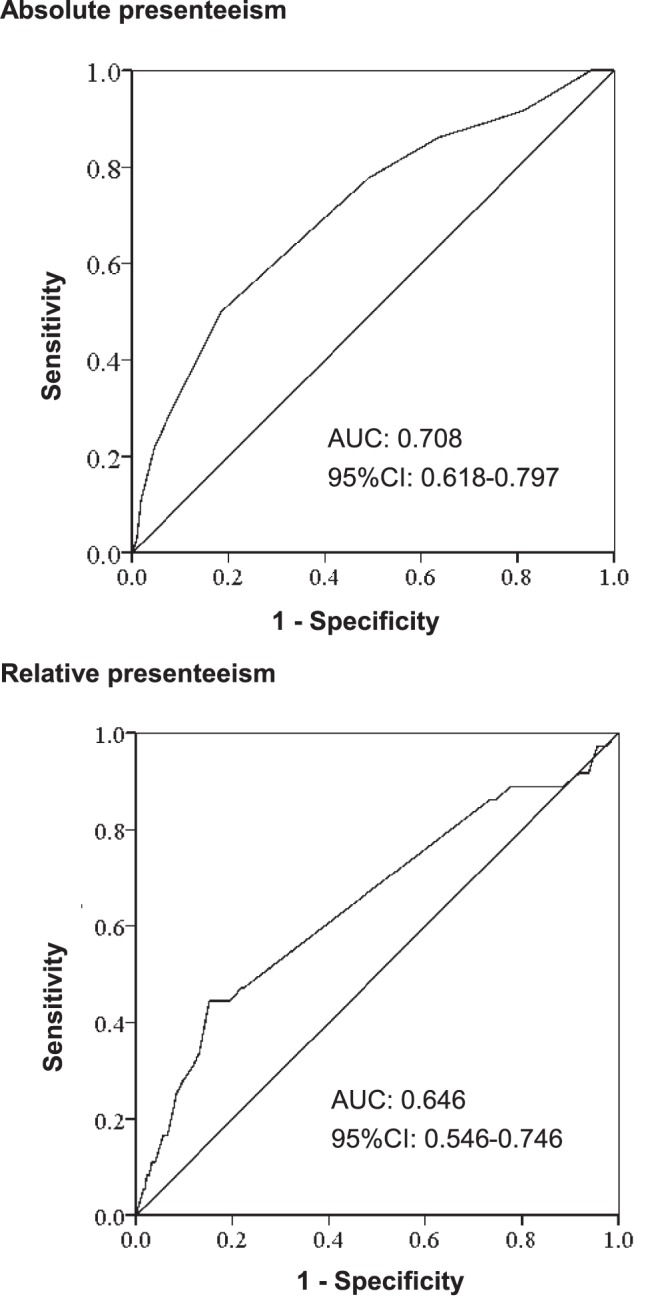
ROC curve and AUC for absolute and relative presenteeism. Receiver-operating characteristic (ROC) analysis of absolute and relative presenteeism as indicators of mental-disease-related absence; AUC: the area under the curve; 95% CI: 95% confidence interval.

The ROC curves for absolute and relative presenteeism score are shown in Fig. 2. The AUC values for absolute and relative presenteeism scores were 0.708 (95% CI, 0.618–0.797) and 0.646 (95% CI, 0.546–0.746), respectively. These data suggest that absolute presenteeism score could be used as a predictor of future absence due to mental disease.

**Figure 3:**
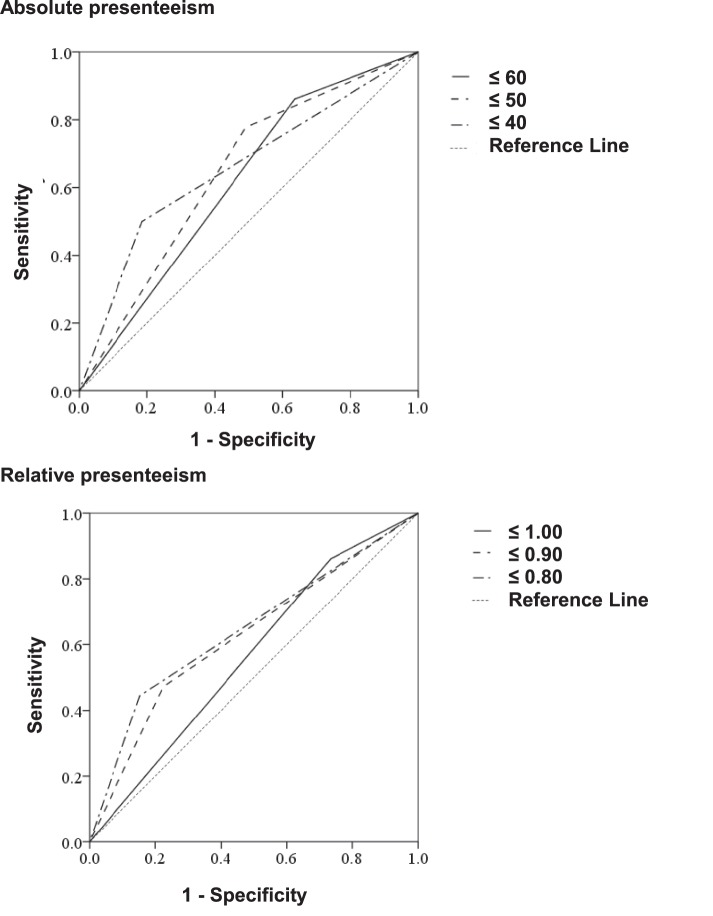
ROC curve of 3 cutoff values for absolute and relative presenteeism. Receiver-operating characteristic (ROC) analysis of absolute and relative presenteeism as indicators of mental-disease-related absence; AUC: the area under the curve; 95%CI: 95% confidence interval; A ROC analysis of absolute presenteeism revealed cutoff points of 40, 50, and 60. A ROC analysis of relative presenteeism revealed cutoff points of 0.8, 0.9, and 1.0.

The sensitivity, specificity, and AUCs of the cutoff scores of absolute and relative presenteeism for the prediction of absence due to mental disease are shown in Table 2 and Fig. 3. In the ROC analyses of predictors of absence due to mental disease, the cutoff point for absolute presenteeism was 40, with a sensitivity of 50.0% and a specificity of 81.4%, and the cutoff point for relative presenteeism was 0.8, with a sensitivity of 44.4% and a specificity of 84.7%. The high specificity is more likely to identify those who do not have the disease, whereas the low sensitivity could underestimate. It would be achieved good specificity and reasonable sensitivity. The values of absolute and relative presenteeism that maximized the Youden index (sensitivity+specificity minus 1) were 40 and 0.8, respectively. This result is consistent with that of Youden index method. These cutoff values were considered optimal according to the sensitivity, specificity, AUC, and the 95% CIs.

**Table 2:** Sensitivity, specificity, and AUC of cutoff value of absolute and relative presenteeism in the prediction of absence due to mental disease.

Indicators	Cutoff value	Sensitivity (%)	Specificity (%)	AUC	95% CI	P
Absolute presenteeism	40	50.0	81.4	0.657	0.558–0.757	0.001*
	50	77.8	50.8	0.643	0.560–0.726	0.003*
	60	86.1	36.4	0.613	0.531–0.694	0.020*
Relative presenteeism	0.8	44.4	84.7	0.646	0.544–0.747	0.003*
	0.9	47.2	77.5	0.624	0.525–0.723	0.011*
	1.0	86.1	26.7	0.564	0.477–0.651	0.187

**Figure 4:**
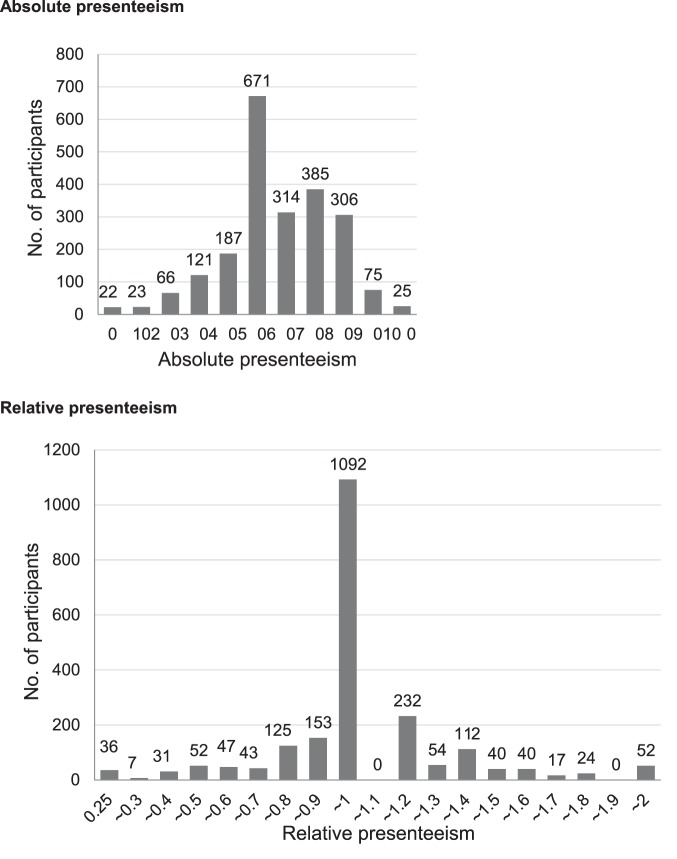
Histograms of absolute and relative presenteeism in our cohort.

AUC: the area under the curve; 95% CI: 95% confidence interval;

Histograms of absolute and relative presenteeism in our cohort are shown in Fig. 4. The greatest number of participants received a score of 50 for absolute presenteeism, and 1.0 for relative presenteeism. There were 419 positive participants (19.1%) with our proposed cutoff score for absolute presenteeism (≤40), and 341 positive participants (15.8%) with our proposed cutoff score for relative presenteeism (≤0.8). No participants scored between 1.0 and 1.1. Relative presenteeism is a ratio of actual performance to the performance of most workers in the same job [14]. The two items of the presenteeism scale were scored using integers from 0 to 10. The smallest value and questionnaire 1 score<questionnaire 2 score was 1.11. Therefore, it is reasonable that no participants scored between 1.0 and 1.1.

### ORs and 95% CIs for sickness absence due to mental disease or non-mental disease according to proposed cutoff score of presenteeism in actual prospective cohort data

ORs and 95% CIs for absence due to mental disease or non-mental disease according to our proposed cutoff score of absolute presenteeism (≤40) in actual prospective cohort data are shown in Table 3. After adjusting for age and gender, participants who scored worse than our proposed cutoff score for absolute presenteeism (≤40) were significantly more likely to be absent because of mental disease (OR = 4.75, 95% CI: 2.17–10.37). In a second model, after adjusting for age, gender, and depressive symptoms (K6≥13) at baseline, these participants were still significantly more likely to be absent (OR = 4.54, 95% CI: 2.07–9.95). In a third model, we performed a multivariate adjustment, but the results were essentially unchanged. On the other hand, participants were more likely to be absent because of non-mental disease after multivariate adjustment (OR = 1.29, 95% CI: 1.01–1.66). Absence due to mental disease had a large effect size, and worse absolute presenteeism scores were consistently and clearly associated with more absences due to mental disease. By contrast, absence due to non-mental disease had a small effect size, and absolute presenteeism scores were not consistently associated with more absences, according to adjustment status.

**Table 3:** Odds ratios and 95% CIs for absence due to mental disease or non-mental disease according to proposed cutoff scores for absolute presenteeism (≤40) in actual prospective cohort data.

	No. ofsubjects	No. ofcases^a^	Absence due to mental disease			No. ofsubjects	No. ofcases^b^	Absence due tonon-mental disease		
			OR	95% CI	P			OR	95% CI	P
Crude	2195	36	4.38	2.26–8.50	<0.001*	2195	821	1.33	1.07–1.66	0.009*
Age- and gender-adjusted	1831	26	4.75	2.17–10.37	<0.001*	1831	670	1.27	0.99–1.62	0.056
Age- gender- and depressive symptom-(K6≥13) adjusted	1830	26	4.54	2.07–9.95	<0.001*	1830	669	1.25	0.98–1.60	0.074
Multivariate-adjusted	1808	26	4.85	2.20–10.73	<0.001*	1808	659	1.29	1.01–1.66	0.042*

a Subjects with absence due to mental disease across a 2-year follow up; ^b^Subjects with absence due to non-mental disease across a 2-year follow up; OR: odds ratio; CI: confidence interval; *P<0.05; adjusted ORs and 95% CIs were based on multiple logistic regression analysis. The first model was adjusted for age and gender, a second model was further adjusted for depressive symptoms (K6≥13) at baseline, and a third model was further adjusted for drinking habits (drink approximately every day or not), smoking habits (current smoker or not), education level (years), job position (managerial job or not), equivalent income (10,000 yen/year), and exercise in spare time (yes or no) at baseline.

ORs and 95% CIs for absence due to mental disease or non-mental disease according to our proposed cutoff score for relative presenteeism (≤0.8) in actual prospective cohort data are shown in Table 4. After adjusting for age and gender, participants who scored worse our proposed cut-off score for relative presenteeism (≤0.8) were significantly more likely to be absent because of mental disease (OR = 5.39, 95% CI: 2.44–11.91). After adjusting for age, gender, and depressive symptoms (K6≥13) at baseline, these participants were also significantly more likely to be absent (OR = 5.47, 95% CI: 2.46–12.15). Again, the results after the multivariate adjustment were essentially unchanged. On the other hand, the participants were significantly more likely to be absent because of non-mental disease after multivariate adjustment (OR = 1.31, 95% CI: 1.01–1.72). Absence due to mental disease had a large effect size, while absence due to non-mental disease had a small effect size.

**Table 4:** Odds ratios and 95% CIs for absence due to mental disease or non-mental disease according to proposed cutoff score for relative presenteeism (≤0.8) in actual prospective cohort data.

	No. ofsubjects	No. ofcases^a^	Absence due tomental disease			No. ofsubjects	No. ofcases^b^	Absence due to non-mental disease		
			OR	95% CI	P			OR	95% CI	P
Crude	2157	36	4.42	2.27–8.62	<0.001*	2157	808	1.42	1.13–1.8	0.003*
Age- and gender-adjusted	1802	26	5.39	2.44–11.91	<0.001*	1802	660	1.32	1.01–1.72	0.040*
Age-, gender-, and depressive symptom-(K6≥13) adjusted	1801	26	5.47	2.46–12.15	<0.001*	1801	659	1.32	1.01–1.71	0.042*
Multivariate-adjusted	1779	26	5.37	2.42–11.93	<0.001*	1779	649	1.31	1.01–1.72	0.046*

a Subjects with absence due to mental disease across a 2-year follow up; ^b^Subjects with absence due to non-mental disease across a 2-year follow up: OR: odds ratio; CI: confidence interval; *P<0.05; adjusted ORs and 95% CIs were based on multiple logistic regression analysis. The first model was adjusted for age and gender, a second model was further adjusted for depressive symptoms (K6≥13) at baseline, and a third model was further adjusted for drinking habits (drink approximately every day or not), smoking habits (current smoker or not), education level (years), job position (managerial job or not), equivalent income (10,000 yen/year), and exercise in spare time (yes or no) at baseline.

ORs and 95% CIs for depressive symptoms (K6≥13) after one year according to our proposed absolute and relative presenteeism scores are shown in Table 5. After adjusting for age and gender, our proposed absolute and relative presenteeism cutoff scores were significantly associated with greater depressive symptoms (K6≥13) after one year (OR = 5.41, 95% CI: 3.73–7.84, and OR = 4.79, 95% CI: 3.25–7.05, respectively). Neither adjusting for age, gender, and depressive symptoms (K6≥13) at baseline nor performing a multivariate adjustment changed the results.

**Table 5:** Odds ratios and 95% CIs for depressive symptoms (K6≥13) after one year according to proposed absolute presenteeism or relative presenteeism cutoff scores.

	No. ofsubjects	No. ofcases^a^	Absolute presenteeism (≤40)			No. ofsubjects	No. ofcases^a^	Relative presenteeism (≤0.8)		
			OR	95% CI	P			OR	95% CI	P
Crude	2195	149	5.33	3.79–7.50	<0.001*	2157	144	5.01	3.52–7.13	<0.001*
Age- and gender-adjusted	1831	128	5.41	3.73–7.84	<0.001*	1802	124	4.79	3.25–7.05	<0.001*
Age-, gender-, and depressive symptom- (K6≥13) adjusted	1830	128	5.20	3.58–7.56	<0.001*	1801	124	4.83	3.27–7.15	<0.001*
Multivariate-adjusted	1808	126	5.19	3.56–7.59	<0.001*	1779	122	4.81	3.24–7.15	<0.001*

a Subjects with a K6 score of ≥13 after one year; OR: odds ratio; CI: confidence interval; *P<0.05; adjusted ORs and 95% CIs were based on multiple logistic regression analysis. The first model was adjusted for age and gender, a second model was further adjusted for depressive symptoms (K6≥13) at baseline, and a third model was further adjusted for drinking habits (drink approximately every day or not), smoking habits (current smoker or not), education level (years), job position (managerial job or not), equivalent income (10,000 yen/year), and exercise in spare time (yes or no) at baseline.

## Discussion

Presenteeism is associated with future sickness absence [9]–[11]. To our knowledge, this is the first study to determine optimal cutoff values of WHO-HPQ presenteeism scores by ROC analysis for the prevention of mental sickness absence. There is no AUC analysis of WHO-HPQ. Our proposed cutoff scores for absolute and relative presenteeism are 40 and 0.8, respectively. The cutoff would be provided the best combination of diagnostic sensitivity and specificity. It would be achieved good specificity, but sensitivity is relatively low. However, the ROC curve displays the trade-off between sensitivity and specificity. Furthermore, when the aim is to evaluate a risk factor, it is preferable to have a test with very high specificity and lower sensitivity, to avoid false positive findings and, consequently, bias in risk estimates [21].

It is very important to develop an effective screening tool for presenteeism. Sickness absence is a public health risk marker for mortality. Melchior et al. reported that psychiatric sickness absence from work appears to be a valid indicator of future mortality risk [22]. Roelen et al. reported that the prior sickness absence episodes model accurately predicted the risk of increased sickness absences in office workers [23], [24].

Various tools have been developed to measure presenteeism. The American College of Occupational and Environmental Medicine Expert Panel has recommended several instruments to measure health-related workplace productivity, such as the WHO-HPQ [25]. One of the most commonly used instruments is the WHO-HPQ, a self-report instrument designed to estimate the consequence of health problems on job performance, illness-related absence, and work-related accidents and injuries [4], [26]. The WHO-HPQ appears to be the instrument best suited for conversion into monetary units [26]. According to the above reports, it was thought that the WHO-HPQ is one of the best measurements to use. Since there was no validated Japanese version of the WHO-HPQ available, we translated it into Japanese and then had it independently back-translated by an American [14].

The relationship between presenteeism and the risk of sickness absence in our study was stronger than that in previous reports (absolute and relative presenteeism after multivariate adjustment, OR = 4.85 and 5.37, respectively, Tables 3 and 4). Bergström et al. reported that sickness presenteeism was a statistically significant risk factor for future sickness absence (relative risk = 1.40) [10]. Hansen et al. reported that participants who had attended work ill more than six times in the year before baseline had a higher risk of sickness absence (Hazard ratio = 1.74) [11]. One possible reason for this difference in findings is that we limited our examination to the effects of presenteeism on mental disease. In contrast, the risk of sickness absence due to non-mental disease in our study was lower than that due to mental disease (absolute and relative presenteeism after multivariate adjustment, OR = 1.29 and 1.31, respectively, Tables 3 and 4) and was closer to the results of previous reports. Taloyan et al. found that participants who reported sickness presenteeism had a higher risk of sickness absence compared to those who did not report sickness presenteeism, and that emotional exhaustion attenuated the ORs to non-significance for both outcomes; this indicates that the health consequences associated with sickness presenteeism are largely related to mental health [9]. However, after adjustment for age, gender, and depressive symptoms (K6≥13) at baseline in our study, worse levels of absolute and relative presenteeism were significantly associated with more absences. On the other hand, after adjusting for age and gender, participants with depressive symptoms (K6≥13) at baseline were significantly more likely to be absent because of mental disease (data not shown). Thus, our findings suggest that the effects of presenteeism on absences may be independent from those of depressive symptoms. The number of participants with absence due to mental disease and a K6 score ≥13 is small. Therefore, another model was performed. The results after adjustment for age, gender, and depressive symptoms (continuous variable) instead of depressive symptoms (K6≥13) were essentially unchanged (data not shown).

Our results show that both sickness absolute and relative presenteeism are significantly associated with future depressive symptoms (K6≥13) (Table 5). Several previous studies have reported a link between worsening presenteeism and increasing depression severity. Holden et al. reported that all health conditions were associated with a greater risk of presenteeism when comorbid with psychological distress, suggesting that psychological distress exacerbates lost productivity [7]. Jain et al. observed a decrease in overall productivity at all levels of depression, and that presenteeism worsened as depression severity increased [8]. Taken together, our findings regarding the relationship between psychological distress and absolute and relative presenteeism correspond with those of previous studies (Table 5).

Recently, the adverse effects of long working hours, including *karoshi* (“death from overworking”), have been a focus of social concern. It has been suggested that working long hours is associated with sickness presenteeism. Böckerman et al. reported that individuals who work full time, regularly work overtime, or have an extended work week are at increased risk of sickness presenteeism [27]. However, our results were different (data not shown). Further studies are needed in order to discuss this matter.

There are several limitations to our study. First, the measurements of presenteeism and depressive symptoms were based on self-reported data from respondents and were consequently subject to recall bias. However, in an effort to reduce recall bias, the recall period was limited to four weeks and 30 days, respectively. In addition, Kessler et al. reported that, with regard to errors in self-reports of work impairments, WHO-HPQ calibration studies showed good concordance of self-reports with payroll records and archival performance ratings by supervisors and peers [4]. These results suggest that any bias in the estimated effects of conditions on work performance is likely to be minimal. Second, our results may be more applicable to men because the number of female participants in our study was relatively small. However, the rates of female participants with and without absences due to mental disease did not differ from those of men. Furthermore, the analyses of outcomes were performed with adjustment for covariates, including gender. Third, since our participants were workers of one large company, the present results may not apply to the general Japanese population. However, the workers were recruited from 12 offices located throughout Japan (from Hokkaido in the far north to Kyushu in the south). Thus, the geographical distribution was reasonably balanced. Finally, we did not adjust for sickness absences in the year prior to baseline in our outcome analyses. However, according to a study by Hansen et al., the association between sickness presenteeism episodes and future sickness absence persists even when controlling for prior sickness absence [10]. In this regard, our analyses were reasonable. Furthermore, this was the first study using WHO-HPQ official Japanese version, and our result has value.

## Conclusions

Our proposed optimal cutoff scores of absolute and relative presenteeism for the prevention of absence due to mental disease in Japanese workers were 40 and 0.8, respectively. Participants who scored worse than the cutoff score for presenteeism were significantly more likely to be absent in future because of mental disease. Our findings suggest that considering presenteeism in the screening of sickness absence due to mental disease would help prevent such an absence. Further studies are needed.

### Ethics approval

The J-HOPE study received ethics approval from the University of Tokyo Ethics Committee and National Center for Global Health and Medicine Ethics Committee.

## References

[1] Henderson M, Glozier N, Holland Elliott K (2005) Long term sickness absence. BMJ 330: 802–803. 15817531 10.1136/bmj.330.7495.802 PMC556060

[2] Järvisalo J, Andersson B, Boedeker W, Houtman I (2005) Mental disorders as a major challenge in prevention of work disability experiences in finland, germany, the netherlands and sweden. Helsinki: KELA, The Social Insurance Institution Finland. Social Security and Health Report 66.

[3] Japan (2013) Summary of long-term sickness absence of national public servant National Personnel Authority. pp. http://www.jinji.go.jp/kisya/1303/1323tyouki.pdf.

[4] Kessler RC, Barber C, Beck A, Berglund P, Cleary PD, et al (2003) The World Health Organization Health and Work Performance Questionnaire (HPQ). J Occup Environ Med 45: 156–174. 10.1097/01.jom.0000052967.43131.51 12625231

[5] Kessler RC, Ames M, Hymel PA, Loeppke R, McKenas DK, et al (2004) Using the World Health Organization Health and Work Performance Questionnaire (HPQ) to evaluate the indirect workplace costs of illness. J Occup Environ Med 46: S23–37. 15194893 10.1097/01.jom.0000126683.75201.c5

[6] Kessler R, Petukhova M, McInnes K (2007) World Health Organization Health and Work Performance Questionnaire (HPQ). HPQ Short Form (Absenteeism and Presenteeism Questions and Scoring Rules) Harvard Medical School.pp. http://www.hcp.med.harvard.edu/hpq/index.php.

[7] Holden L, Scuffham PA, Hilton MF, Ware RS, Vecchio N, et al (2011) Health-related productivity losses increase when the health condition is co-morbid with psychological distress: findings from a large cross-sectional sample of working Australians. BMC Public Health 11: 417. 21627840 10.1186/1471-2458-11-417 PMC3129311

[8] Jain G, Roy A, Harikrishnan V, Yu S, Dabbous O, et al (2013) Patient-reported depression severity measured by the PHQ-9 and impact on work productivity: results from a survey of full-time employees in the United States. J Occup Environ Med 55: 252–258. 23439268 10.1097/JOM.0b013e31828349c9

[9] Taloyan M, Aronsson G, Leineweber C, Magnusson Hanson L, Alexanderson K, et al (2012) Sickness presenteeism predicts suboptimal self-rated health and sickness absence: a nationally representative study of the Swedish working population. PLoS One 7: e44721. 22984547 10.1371/journal.pone.0044721 PMC3439368

[10] Hansen CD, Andersen JH (2009) Sick at work-a risk factor for long-term sickness absence at a later date? J Epidemiol Community Health 63: 397–402. 19366890 10.1136/jech.2008.078238

[11] Bergstrom G, Bodin L, Hagberg J, Aronsson G, Josephson M (2009) Sickness presenteeism today, sickness absenteeism tomorrow? A prospective study on sickness presenteeism and future sickness absenteeism. J Occup Environ Med 51: 629–638. 19448572 10.1097/JOM.0b013e3181a8281b

[12] Miyaki K, Song Y, Htun NC, Tsutsumi A, Hashimoto H, et al (2012) Folate intake and depressive symptoms in Japanese workers considering SES and job stress factors: J-HOPE study. BMC Psychiatry. 12: 33. 10.1186/1471-244X-12-33 PMC3439709 22521003

[13] Suzuki T, Miyaki K, Tsutsumi A, Hashimoto H, Kawakami N, et al (2013) Japanese dietary pattern consistently relates to low depressive symptoms and it is modified by job strain and worksite supports. J Affect Disord 150: 490–498. 23759276 10.1016/j.jad.2013.04.044

[14] Kessler R, Petukhova M, McInnes K (2007) World Health Organization Health and Work Performance Questionnaire (HPQ). Japanese version of the HPQ Short Form (Absenteeism and Presenteeism Questions and Scoring Rules) Harvard Medical School. pp. http://www.hcp.med.harvard.edu/hpq/info.php.

[15] Furukawa TA, Kawakami N, Saitoh M, Ono Y, Nakane Y, et al (2008) The performance of the Japanese version of the K6 and K10 in the World Mental Health Survey Japan. Int J Methods Psychiatr Res 17: 152–158. 18763695 10.1002/mpr.257 PMC6878390

[16] Kessler RC, Andrews G, Colpe LJ, Hiripi E, Mroczek DK, et al (2002) Short screening scales to monitor population prevalences and trends in non-specific psychological distress. Psychol Med 32: 959–976. 12214795 10.1017/s0033291702006074

[17] Sakurai K, Nishi A, Kondo K, Yanagida K, Kawakami N (2011) Screening performance of K6/K10 and other screening instruments for mood and anxiety disorders in Japan. Psychiatry Clin Neurosci 65: 434–441. 21851452 10.1111/j.1440-1819.2011.02236.x

[18] Kessler RC, Barker PR, Colpe LJ, Epstein JF, Gfroerer JC, et al (2003) Screening for serious mental illness in the general population. Arch Gen Psychiatry 60: 184–189. 12578436 10.1001/archpsyc.60.2.184

[19] Boyd J, editor (2007) Statistical Analysis and Presentation of Data Evidence-Based Laboratory Medicine; Principles, Practice and Outcomes. 2. Washington DC, USA: AACC Press. 113–140 p.

[20] Florkowski CM (2008) Sensitivity, specificity, receiver-operating characteristic (ROC) curves and likelihood ratios: communicating the performance of diagnostic tests. Clin Biochem Rev 29 Suppl 1: S83–87. 18852864 PMC2556590

[21] Copeland KT, Checkoway H, McMichael AJ, Holbrook RH (1977) Bias due to misclassification in the estimation of relative risk. Am J Epidemiol 105: 488–495. 871121 10.1093/oxfordjournals.aje.a112408

[22] Melchior M, Ferrie JE, Alexanderson K, Goldberg M, Kivimaki M, et al (2010) Does sickness absence due to psychiatric disorder predict cause-specific mortality? A 16-year follow-up of the GAZEL occupational cohort study. Am J Epidemiol 172: 700–707. 20732935 10.1093/aje/kwq186 PMC2938268

[23] Roelen CA, Bultmann U, van Rhenen W, van der Klink JJ, Twisk JW, et al (2013) External validation of two prediction models identifying employees at risk of high sickness absence: cohort study with 1-year follow-up. BMC Public Health 13: 105. 23379546 10.1186/1471-2458-13-105 PMC3599809

[24] Roelen CA, van Rhenen W, Groothoff JW, van der Klink JJ, Bultmann U, et al (2013) The development and validation of two prediction models to identify employees at risk of high sickness absence. Eur J Public Health 23: 128–133. 22539631 10.1093/eurpub/cks036

[25] Lack DM (2011) Presenteeism revisited. A complete review. AAOHN J 59: 77–89; quiz 90–71. 10.3928/08910162-20110126-01 21323209

[26] Despiegel N, Danchenko N, Francois C, Lensberg B, Drummond MF (2012) The use and performance of productivity scales to evaluate presenteeism in mood disorders. Value Health 15: 1148–1161. 23244819 10.1016/j.jval.2012.08.2206

[27] Bockerman P, Laukkanen E (2010) What makes you work while you are sick? Evidence from a survey of workers. Eur J Public Health 20: 43–46. 19525328 10.1093/eurpub/ckp076

